# Genetic conflicts and the case for licensed anthropomorphizing

**DOI:** 10.1007/s00265-022-03267-6

**Published:** 2022-12-01

**Authors:** J. Arvid Ågren, Manus M. Patten

**Affiliations:** 1 Department of Evolutionary Biology, Uppsala University, Uppsala, Sweden; 2 Present Address: Lerner Research Institute, Cleveland Clinic Foundation, Cleveland, USA; 3 Department of Biology, Georgetown University, Washington, DC USA

**Keywords:** Gene’s-eye view of evolution, Selfish genetic elements, Genomic imprinting, X chromosomes

## Abstract

The use of intentional language in biology is controversial. It has been commonly applied by researchers in behavioral ecology, who have not shied away from employing agential thinking or even anthropomorphisms, but has been rarer among researchers from more mechanistic corners of the discipline, such as population genetics. One research area where these traditions come into contact—and occasionally clash—is the study of genetic conflicts, and its history offers a good window to the debate over the use of intentional language in biology. We review this debate, paying particular attention to how this interaction has played out in work on genomic imprinting and sex chromosomes. In light of this, we advocate for a synthesis of the two approaches, a form of licensed anthropomorphizing. Here, agential thinking’s creative potential and its ability to identify the fulcrum of evolutionary pressure are combined with the rigidity of formal mathematical modeling.

## Introduction

A major achievement of modern social evolution theory is the expansion of its purview. We now appreciate that conflict and cooperation happen at all levels of life—from cells to societies (Maynard Smith and Szathmáry 1995; Queller 1997; Michod 1999; Bourke 2011; Foster 2011). This development has revealed deep parallels between systems of different scales, such as the importance of high degrees of relatedness in the evolution of both multicellularity and eusociality (West et al. 2015) and the key role of enforcement mechanisms in minimizing the negative effects of conflicts (Ågren et al. 2019a). It has also led to the application of approaches first developed with whole organisms in mind to new domains.

One such approach is agential thinking, where it is typically organisms that are conceptualized as agents pursuing goals and strategies (Okasha 2018; Veit 2021). In addition to playing an important role in animal behavior research (Davies et al. 2012; West and Gardner 2013), biologists have applied agential thinking to entities other than organisms. One example is the study of genetic conflicts, where the fitness interest of genes of the same organism diverge (Burt and Trivers 2006; Werren 2011; Ågren and Clark 2018). The evolution of such conflicts has attracted a broad spectrum of researchers, including molecular biologists, theoretical population geneticists, and behavioral ecologists. These sub-disciplines vary in how comfortable they are with applying intentional language to genes. Behavioral ecologists, with their long tradition of modeling organisms as agents behaving as if trying to maximize their (inclusive) fitness, have had little issue with applying similar methods to genes. In contrast, molecular biologists and population geneticists have rejected such approaches in favor of more mechanistic explanations.

An illustrative example of this clash occurred in 2006 when Austin Burt and Robert Trivers published the majestic *Genes in Conflict* (Burt and Trivers 2006). The first book-length treatment of the biology of selfish genetic elements, genes that enhance their own evolutionary success at the expense of other genes, the volume was extremely well-received, with numerous favorable reviews in venues such as *Nature* (Crow 2006), *Nature Genetics* (Malik 2007), and *Science* (Hammerstein and Hagen 2006). In *Current Biology*, the population geneticist Brian Charlesworth praised the book but also noted that:

My only serious complaint is that the book is light on the theory underlying the interpretations that are offered. The language is often *surprisingly anthropomorphic*. (…) Genes or genetic elements do not “want” anything: evolution is a purely mechanistic process of shifts in the frequencies of genetic variants of one kind or another.

—B. Charlesworth (2006; our emphasis).

We pick up on Charlesworth’s concern and discuss its implications for the debate over intentional language in biology. We first review the biology of genetic conflicts and its connection to the so-called gene’s-eye view of evolution, a perspective that grew out of behavioral ecology and locates evolutionary agency at the level of the gene rather than the organism. Next, we use examples from genomic imprinting and sex chromosome conflicts to discuss the criticism that this connection has been subject to, and how that reveals a division between researchers who approach the topic from a behavioral ecology background, as opposed to that of theoretical population genetics. In light of this, we make the case for a form of licensed anthropomorphizing. Here, agential thinking helps generate hypotheses and identify the site of selective action, insights that are then backed up by, and grounded in, formal population genetic modeling.

## Genetic conflicts and organismal unity of purpose

The genome is an extraordinary unit of cooperation. Most traits, in most organisms, are the product of the coordinated effort of multiple genes. In fact, cooperation between genes usually works so well that biologists take it for granted (Doolittle and Sapienza 1980; Orgel and Crick 1980; Dawkins 1990). However, there are many ways that genic cooperation breaks down (Burt and Trivers 2006; Werren 2011; Ågren and Clark 2018). In particular, selfish genetic elements come into conflict with the rest of the genome by, for example, subverting the normal rules of replication and inheritance to promote their own transmission. Selfish genetic elements exist in a wide diversity of flavors: segregation distorters and meiotic drivers interfere with the process of meiosis and end up in more than 50% of all the gametes (Larracuente and Presgraves 2012; Lindholm et al. 2016); homing endonucleases convert heterozygotes to homozygotes and so guarantee their own transmission (Burt and Koufopanou 2004; Oberhofer et al. 2018); transposable elements self-replicate and then insert into new locations in the genome (Tenaillon et al. 2010; Kapusta et al. 2017). Other genetic conflicts arise because genes differ in whether they are inherited through both sexes or exclusively through one (Lewis 1941; Frank and Hurst 1996).

Genetic conflicts are ever-present in sexually reproducing eukaryotes, and their abundance has many methodological implications. Take for example the popular approach where evolved entities are conceptualized as agents pursuing goals (reviewed in Okasha 2018). The quintessential agent is the organism, which, in many ways, is common sense: it is organisms that struggle to survive and organisms that compete to reproduce, and so the organism is regarded as the entity of adaptation. However, as noted by Okasha (2018, pp. 28–34), a strict organismal account of agency depends on an assumption of a within-body unity of purpose (see also Dawkins 1982, p. 134; Dawkins 1990; West and Gardner 2013). That is, all parts of the organism work together for the same goal: to enhance the (inclusive) fitness of the individual. But as we have seen, not all parts of organisms actually work together. Instead, the organism is constantly threatened from within. Individual-centric models of adaptation may therefore struggle to accommodate genetic conflicts, and biologists have looked for other frameworks to help them make sense of them. Leading among those is the gene’s-eye view of evolution (Haig 2014a; Ågren 2016).

## The gene’s-eye view of evolution and the Oxford school of biological science fiction

If we allow ourselves the license of talking about genes as if they had conscious aims, always reassuring ourselves that we could translate our sloppy language back into respectable terms if we wanted to, we can ask the question, what is a single selfish gene trying to do?

—R. Dawkins (1976, p. 88)

So asks Richard Dawkins in *The Selfish Gene,* and the question is a pithy expression of the gene’s-eye view of evolution (Ågren 2021). The gene’s-eye view is a way to think about evolution and natural selection that was first laid out by George Williams in *Adaptation and Natural Selection* (Williams 1966; see also Williams 1992) and later by Dawkins (1976, 1982). The approach locates agency at the genetic rather than organismal level, by combining the insight from population genetics that evolution can be described as a change in allele frequencies with the agential thinking of behavioral ecology. The central reason for this transfer of agency is that only genes are transmitted from parent to offspring. Organisms and their phenotypes, in contrast, are destroyed every generation. Genes, the argument goes, are the ultimate beneficiaries of natural selection.

Under the gene’s-eye view of evolution, intentionality is used in an as-if manner. As Dawkins emphasized in *The Selfish Gene*:

we must not think of genes as conscious, purposeful agents. Blind natural selection, however, makes them behave rather as if they were purposeful, and it has been convenient, as a shorthand, to refer to genes in the language of purpose. For example, when we say ‘genes are trying to increase their numbers in future gene pools’, what we really mean is ‘those genes that behave in such a way as to increase their numbers in future gene pools tend to be the genes whose effects we see in the world’.

—R Dawkins 1976, p. 196

Although its heuristic use may seem obvious—because no sane person thinks DNA molecules have conscious personalities (Dawkins 2016, p. viii)—the gene’s-eye view’s habit of anthropomorphizing genes has provoked some excellent insults. Some of our personal favorites include Rosenberg’s “conspiracy theorists” (Rosenberg 2011, pp. 13–14) and Francis’s and Godfrey-Smith’s “Darwinian paranoia” (Francis 2004, p. 8; Godfrey-Smith 2009, p. 10). More generally, many have warned about the dangers of agential thinking and of anthropomorphizing. For example, in *Biological Science*, a widely used introductory textbook in its time, William Keeton wrote that we “must constantly guard against unwarranted attribution of human characteristics to other species. Anthropomorphic or teleological thinking has no place in a scientific study” (Keeton 1967, p. 425). Half a century later, Peter Godfrey-Smith described agential thinking as a “trap” that has:

real heuristic power in some contexts, but also has a strong tendency to steer us wrongly, especially when thinking about foundational issues. And once we start thinking in terms of little agents with agendas—even in an avowedly metaphorical spirit—it can be hard to stop.

—P. Godfrey-Smith (2009, p. 5)

Lucy Sullivan attributed the gene’s-eye view to what she dubbed the “Oxford school of biological science fiction” (Sullivan 1995). Her paper is an especially fierce version of the critique of agential thinking at the genic level and she opens her argument by describing the gene’s-eye view as having “achieved a hegemony quite out of proportion to its intellectual finesse” and its anthropomorphism as an “infection” spreading through the body of biology with increasing vigor:

…for close to a century, biology as a discipline managed to indoctrinate its students with this care [to avoid anthropomorphizing] that is essential to its *scientific purity*. It was kept clean of anthropomorphism by constant mutual vigilance.

—L. Sullivan (1995; our emphasis)

The paper that had defiled scientific purity in such a way as to prompt Sullivan’s attack was one on the evolution of sex written by Laurence Hurst and W.D. Hamilton. In the offending paper, Hurst and Hamilton (1992) propose a novel answer to the question: why are there separate sexes? Their solution (“fable”, as Sullivan calls it) is that separate sexes/binary mating types arise as a way to avoid genetic conflict. They argue that when two cells fuse during sex there is a risk of conflict between the two sets of organelles, and it is in response to this risk that one sex (typically males) have evolved to not transmit their organellar genome(s). In support of this hypothesis, they first present a mathematical model that shows that if a gene in the nuclear genome is able to destroy its own mitochondrial genome upon transmission, it will invade a population if it is simultaneously linked to a locus that makes it mate with the opposite mating type. Next, they show that sexually reproducing organisms come in two main groups, those that fuse cells and those that do not, and that it is only in the fusing group that you find separate sexes. Only then is there an expectation of organellar conflict. In the non-fusing group, only the nuclei are exchanged, and the opportunity for conflict does not present itself.

In her critique, Sullivan primarily targets a News and Views-style perspective that covered the Hurst and Hamilton paper (Anderson 1992). She decries the lack of what she calls neutral words (she offers “competition” and “incompatibility” as examples of such neutrality) and objects to the use of terms like “murderous mitochondrion”, descriptions of the “unilateral disarmament” and “surrender” of organelles, and the risk of “war breaking out between two sets of organelles”. Unfortunately, Sullivan’s description of the science at hand reveals some serious confusions. She mischaracterizes the Hurst and Hamilton model, David Haig’s kinship theory of genomic imprinting (which she describes as a “breath-taking leap”), as well as both *The Selfish Gene* and *The Origin of Species* (see Dawkins 1995).

Nevertheless, hidden underneath the amped-up rhetoric in Sullivan’s paper, is a fair point about the use of agential thinking and the accompanying anthropomorphic language in the study of genetic conflicts. She notes that such language may distract rather than contribute to an understanding of their biology. This is not a critique to dismiss lightly. Biology, and especially the branch of the discipline dedicated to studying animal behavior, has wrestled with it for decades (for overviews see, for example, Mitchell et al. 1997; Crist 1999; Daston and Mittman 2005). Several influential names in ethology—in many ways the predecessors of behavioral ecology and, to a lesser extent, sociobiology (Stuhrmann 2022)—addressed the topic numerous times. This included Niko Tinbergen (1963), Konrad Lorenz (who dealt with the topic in his Nobel lecture; 1974), and, in particular, J.S. Kennedy (1954, 1992). Tinbergen, Lorenz, and Kennedy wrote from within ethology and sought to improve its scientific standing as a field. Because we are concerned with how this criticism played out in the research on genetic conflicts, we now turn to another corner of biology that has influenced the debate over anthropomorphism and our understanding of genetic conflicts: theoretical population genetics. To illustrate the debate between on the one hand the behavioral ecology tradition, with its anthropomorphic agential thinking, and on the other formal population genetic approaches, which steer clear of agency entirely, we will consider two examples: genomic imprinting and sex chromosomes.

## Example 1: Genomic imprinting

For most genes in a diploid organism, we expect similar levels of expression from both alleles. In a few hundred genes in mammalian and seed-plant genomes, however, we find that expression differs between the two alleles—often a difference of silence vs. expression—in a parent-specific fashion (Bartolomei and Ferguson-Smith 2011; Babak et al. 2015; Picard and Gehring 2020). These “imprinted genes” were first uncovered in mammals in the 1980s and 1990s (reviewed in Ferguson-Smith 2011), and almost immediately they raised questions for evolutionary biologists. Chief among them: why did such parental-origin-specific, or imprinted, expression evolve?

Evolutionary geneticists have gone about answering this question in different ways. For those coming from the behavioral ecology tradition, the key to understanding the origin of imprinted expression is to provide an account of the adaptive benefit of this unique form of gene expression and to identify who exactly benefits. In his kinship theory of the evolution of genomic imprinting, David Haig draws on this tradition and locates the adaptive benefit for imprinted expression in a non-traditional place (Haig 2000, 2002). Whereas the typical behavioral ecology model involves individual organisms pursuing a goal of inclusive-fitness maximization, Haig, taking a gene’s-eye view, instead lets each allele at a diploid locus pursue its own inclusive fitness maximization. Haig’s crucial insight was recognizing that diploid individuals are related to most of their social partners differently for their matrigenic (maternally derived) and patrigenic (paternally derived) genes. Haig (2014b) employs agential thinking and language in his description of his logic:

Imprinted expression can be viewed as a conditional strategy of an allele that adopts matrigenic and patrigenic roles in different rounds of an evolutionary tournament. Natural selection favors imprinted expression when conditional (imprinted) strategies outperform unconditional (unimprinted) strategies.

—D. Haig (2014b)

For example, consider a gene whose expression promotes fetal growth by soliciting maternal resources during a mammalian pregnancy. A lower expression level of this gene would be favored when maternally derived than paternally derived because excessive demands made by the growing fetus on its mother will decrease the gene’s matrilineal inclusive fitness (via the loss of future maternal sibs), but patrilineal inclusive fitness may be virtually unaffected—or even enhanced—if future siblings are maternal half-sibs sired by other males. Using a game theoretic model, Haig demonstrated that silencing of one allele at a locus and expression from the other is the evolutionary stable strategy (ESS) of a gene that meets these assumptions (Haig 1997; reviewed in Wilkins and Haig 2003). By and large, the empirical data fit neatly with Haig’s theory (Haig 2004; Moore and Mills 2008; Patten et al. 2014).

For evolutionary geneticists in the population genetic tradition, the above account has been unsatisfying. To start, behavioral ecologists and population geneticists have rather different comfort levels with agential thinking. Take, for example, this comment from the population geneticist Marc Feldman:

I don’t care whether you talk about game theory in evolution or inclusive fitness or maximization. This line of thinking that animals sit there optimizing things is foreign to population genetics.

—M. Feldman (quoted in Schwartz 2002).

Further, the adoption of an ESS approach commits one to search for some stable state that the system will evolve to, a point where an individual’s (inclusive) fitness is maximized and cannot be further increased by any shift in strategy (Lehmann and Rousset 2020). Ever since Moran’s (1963) demonstration that mean fitness can decrease in a purely deterministic model, population geneticists have been uneasy arguing from maxima (see Grodwohl 2017 for a historical overview).

Another issue is that the details of the genetics are completely ignored in a game theoretic model. Presumably, between an unimprinted state and an imprinted state there are intermediate stages—or, at least, there could be, given that a wholly new expression strategy might not emerge in a single mutational step—but in a game theoretic approach one will not find any mention of these alleles. Similarly, details of dominance, epistasis, and effect size are omitted. These genetic considerations could also, in principle, prevent individuals and populations from achieving some fitness maximum.

At the heart of the issue is a disagreement over what a satisfactory explanation looks like. To a population geneticist, there can be no answer to why imprinting evolves until an explicit, formal population-genetic model has been constructed. For them, this is the gold standard method for validating any evolutionary hypothesis. And indeed, population genetic models have confirmed the insight that imprinting can evolve when relatedness to social partners differs for matrigenic and patrigenic alleles (e.g., Spencer et al. 1998; reviewed in Spencer et al. 1999; Spencer 2000). But along with that confirmation, we also find one of the challenges that population genetic models—particularly models of social interactions—face. One should avoid crafting models that are as complex as nature and should retain only the essential elements, but to do so for the case of imprinting involves making quite narrow assumptions about social structure and genetics (for example, each female is mated by two males, and genotypes show either bi-allelic or imprinted expression; Spencer et al. 1998). The conflict Haig (1997) identified between alleles at a locus, which he termed “parental antagonism”, is simply not readily captured in such a standard population genetic model (for an exception that strikes a balance between simplicity and realism well, see Brandvain 2010).

For the study of genomic imprinting, the behavioral ecology tradition trades genetic simplicity for social complexity and profits in two ways: it correctly identifies parental antagonism between the alleles at a diploid locus as the source of evolutionary pressure and it arrives at novel and specific predictions that have been largely upheld. The population genetic approach, although useful in validating the former, is forced into assumptions about genetics and social structure that lose sight of where selection is really operating and ends up discussing imprinting as just an exceptional kind of dominance modification with individual-level fitness consequences (Box 1).

**BOX 1:** Choices in how to model sexual and parental antagonism on sex chromosomes

When modeling evolutionary conflict, evolutionary geneticists choose between traditions that emphasize either the agendas of conflicting parties or the genetic details of the biological system. Because there has been no third tradition that allows us to train a clear lens on all of the selective angles while simultaneously preserving a focus on the particulars of the genetics, we advocate a form of pluralism, where conflict is modeled in both ways to harness the unique strengths of each.	
As an illustration, consider Haig (2006) and Patten and Haig (2009). In the former, Haig neatly reasons that matrigenic interests are preferentially served by X chromosomes when there is parentally antagonistic selection. That is, when alleles confer benefits on matrilineal inclusive fitness and harm on patrilineal inclusive fitness or vice versa. Haig arrives at this prediction in a manner similar to how others have predicted a female bias on the X when it comes to sexually antagonistic selection. He reasoned that because the X chromosome will be maternally derived twice as often as paternally derived, genes on the X will be selected more strongly when in the maternally-derived class and so the X chromosome should evolve effects that benefit matrilineal inclusive fitness at the expense of patrilineal inclusive fitness, where all relatednesses are computed in reference to the maternally or paternally derived allele at a locus (Haig 2006).	
Left uncertain by this approach, however, is whether a population will actually evolve to meet this prediction. Might it get hung up on an internal polymorphism instead? And does it matter how strong the selective differences among alleles are? From Haig’s (2006) model, it is clear what X chromosomes “want” to do, but whether genetic particulars will get in the way and prevent them from getting what they want is best tested by population genetic models.	
Patten and Haig (2009) produce such a test with their population genetic model of parental antagonism on the X chromosome. To avoid the unwieldiness that comes with trying to capture social complexities in a population genetic model, they adopt a seemingly odd assumption about the fitness of reciprocal heterozygotes. Specifically, they “captured” parental antagonism by setting reciprocal heterozygotes as the most and least fit female genotypes, respectively (see also Patten et al. 2013).	
This theoretical contrivance ensures that one allele fares better when maternally derived, and vice versa for the other when paternally derived. Because this is not an inclusive fitness scheme, it misrepresents the true causal structure of Haig’s (2006) system, but it nonetheless captures the direction of the forces at play and allows predictions about which sorts of alleles—matrilineal-benefitting or patrilineal-benefitting—have an easier time of invading a population when rare.	
In this case, the two approaches arrive at the same prediction. Modeled either way, the X chromosome has a matrilineally-biased agenda, and alleles that benefit matrilines should therefore come to be over-represented on the X chromosome. Yet, the benefits of pluralism are there to see. From the more agential models, one can clearly identify where selection is operating—who benefits, who suffers the costs—and see intuitively how selection can spur evolutionary change. And from the population genetic models, one comes to trust that details of the genetics do not get in the way of achieving the predicted result from the agential models.	
As a second illustration, we turn to sexual antagonism on the X chromosome where again the two approaches have been profitably combined (see Rice 1984; Patten and Haig 2009; Fry 2010; Frank and Crespi 2011; Mullon et al. 2012; Patten 2019; Frank and Patten 2020)—sometimes even in the same paper (e.g. Hitchcock and Gardner 2020; Klein et al. 2021). The question here is how sexually antagonistic selection will shape genes residing on X chromosomes. Should we expect these genes to be biased in favor of producing male-beneficial or female-beneficial effects? Or unbiased? The early intuition was that owing to the X chromosome spending 2/3 of its time in female bodies, it should achieve a female bias. This intuition has roots in the theory of reproductive value (Frank and Crespi 2011). But with a population genetic approach Patten (2019) showed the possibility that the X chromosome should actually be male-biased in its effects, despite the extra time it spends in females. To arrive at this, he took the standard approach of specifying genotypes and their corresponding fitnesses and then he solved for the conditions that allowed for invasion of novel mutant alleles.	
Specifically, he compared two models (Table 1). These differ in which sex receives the benefit of the mutant allele, *X*_1_, but were taken to be otherwise comparable, provided dominance, *h*_1_, was equal to 1–*h*_2_, this latter assumption made to equalize the dominance of the beneficial allele in the ensuing comparison. Patten (2019) then showed that the first model, in which males gain the benefit of the mutant allele, yielded invasion conditions that were easier to satisfy than the second. Further, when polymorphic equilibria were achieved, Model 1 had higher allele frequencies for *X*_1_ than Model 2.	
When two approaches to the same problem reach different results, there is cause for concern. Here, the resolution of the disagreement is found in the details of the genetics, which are the focus of population genetic models. The two models from Table 1 can only be taken as comparable if we assume that dosage compensation is complete. Note that in Model 1 a single *X*_1_ allele confers a benefit of + *S* on males, but in Model 2 it requires a double dose of *X*_1_ alleles to achieve the same. If we were to do away with that equivalence and instead assume that males, with their lone copy of *X*_1_, were, in terms of fitness, equivalent to female heterozygotes, who also have just the one copy of *X*_1_, then the results from the population genetic analysis would be quite different. As Frank and Patten (2020) showed, it could even yield a female bias.	
The benefits of pluralism are again on display. The agential way of reasoning helps to identify what Hitchcock and Gardner (2020) refer to as the “agenda” of the X chromosome. It is correct to infer that the X chromosome wants to push phenotypes closer to the female optimum. But whether the X chromosome achieves this comes down to its “power” to do so, which power depends on the details of the genetics. From the population genetic approach, we can see clearly how different assumptions about dosage compensation shape the power dynamics between the sexes and how they influence the prediction of any bias on the X chromosome.

**Table 1:** Two models of sexual antagonism on the X chromosome. The two models differ in which sex receives the selective benefit, measured by selection coefficient *S* (*S* > 0), of the rare allele and which suffers the cost, measured by selection coefficient *T*. Dominance is captured by *h*, and it is assumed that *h*_1_ = 1 − *h*_2_

	Genotypes				
	Females			Males	
	*X*_1_*X*_1_	*X*_1_*X*_2_	*X*_2_*X*_2_	*X*_1_*Y*	*X*_2_*Y*
	Fitness values				
Model 1	1 − *T*	1 − *h*_1_*T*	1	1 + *S*	1
Model 2	1 + *S*	1 + *h*_2_*S*	1	1 − *T*	1

This is not to say that population genetic models are, in general, deficient in generating predictions or that they can only serve a confirmatory role in the study of genetic conflicts, as our next example makes clear.

## Example 2: X chromosomes

Unlike autosomal genes, which are transmitted in a symmetric fashion from parents of both sexes to offspring of both sexes, genes on sex chromosomes are transmitted in an asymmetric manner. Such transmission differences can spur the evolution of sex-preferential effects for genes that would experience opposing selection pressure in the two sexes. For example, Y chromosomes, which are passed patrilineally, are predicted to harbor genetic effects that benefit males (Rice 1998; Ågren et al. 2019b). Like Y-linked genes, mitochondrial genes are uniparentally inherited (in most species, most of the time, matrilineally). A crucial difference is that they appear in both sexes, which means that mitochondria are expected to evolve genetic effects that benefit female bearers at the expense of male bearers, a phenomenon that goes by the name “Mother’s Curse” (Frank and Hurst 1996; Gemell et al. 2004). The X chromosome, whose transmission genetics is more tortuous than the above examples, gives the impression that it, too, should develop a preference for one sex. But identifying the direction of that preference and its cause (or causes) has been challenging, as different traditions make different assumptions, both explicitly and implicitly, and at times arrive at opposite predictions.

Again, let us first consider the behavioral ecology tradition. Evolutionary geneticists in this mold, with their fondness for agential thinking and principled neglect of the details of the genetics, approach the question of how the X chromosome should evolve by asking what a gene on the X chromosome wants. Recognizing that 1/3 of the time it would want to produce a male-beneficial phenotype and 2/3 of the time a female-beneficial phenotype (assuming a 1:1 sex ratio), their conclusion is that the X should evolve to produce a phenotype that is, on average, more beneficial to females than to males. This intuitive version of their reasoning rests firmly on the theory of reproductive value, leading to a prediction that the X chromosome will have a female-biased “agenda” (Frank and Crespi 2011; Hitchcock and Gardner 2020).

On the other hand, population geneticists, uncomfortable as they are with agential thinking and primarily concerned with details of the genetics, take a different tack. For the population geneticist, the job is to build a mathematical model, comprising a specified number of genotypes and their associated fitness values, for which invasion conditions and equilibrium frequencies can be derived. No wants or goals need to be invoked, just genotypes reproducing according to the specified rules. The evolutionary prediction is found at the equilibrium. This was the approach taken by Patten (2019) in his study of sexual antagonism on the X chromosome, which led him to a result contradictory to the behavioral ecology logic (Box 1). He showed that on the X chromosome it was easier, all things equal, for alleles that moved a phenotype toward the male optimum rather than the female optimum to invade a population. Furthermore, after having invaded, those male-beneficial alleles reached higher equilibrium frequencies than would equivalent female-beneficial alleles. In a follow-up, Frank and Patten (2020) dissected this result to show which details of the genetics were key to these predictions. Specifically, they showed that Patten’s (2019) “all things equal” assumption implied dosage compensation, which, if relaxed, could tip the bias in the female direction.

The lesson that should be taken from the above studies was best captured by Hitchcock and Gardner (2020), who took a gene’s-eye view of sexual antagonism on the X chromosome. They identified two truths about its response to sexually antagonistic selection. First, one can show by an appeal to reproductive values that the X chromosome, owing to its transmission genetics, does indeed have a female-biased agenda. But second, whether X chromosomes can deliver on that agenda depends on their power to shape phenotypes, and this depends in turn on the assumptions about gene action. That is, for the case of sex biases on the X chromosome, a reliance on behavioral ecology’s tradition of agential thinking alone is insufficient; here, the details of the genetics matter greatly to the outcome. Whereas for imprinting, the behavioral ecology approach was key to arriving at the right causal understanding of the evolved pattern, for X chromosomes, the population genetic models are essential for achieving the same.

In general, agential thinking is an unrivaled way to identify the fulcrum of evolutionary pressure—where the selective action is—but often achieves this by ignoring genetic details. Constructing population genetic models, however, forces us to be explicit about such assumptions. In the study of genetic conflicts, the relative power of the two approaches will often hinge on the importance of those genetic details.

## Charlesworth’s concern revisited: in defense of a licensed anthropomorphism

Let us now return to Charlesworth’s concern: evolution is a purely mechanistic process that has no use for agential or anthropomorphic explanations (Charlesworth 2006). Why then, as David Haig once asked, should we “use strategic thinking, which anthropomorphizes genes, instead of the well-developed infrastructure of population genetics?” (Haig 1997, p. 285). This is a debate that runs through all of evolutionary biology but, as illustrated here, very much comes to the fore in the study of genetic conflicts. The disagreements over how to best conceptualize genomic imprinting and sex chromosome-induced conflicts demonstrate that both anthropomorphic agential thinking and population genetic approaches have things to offer.

Formal population genetic models force us to be explicit about our assumptions. The logic of evolutionary arguments can be deceivingly simple. Problems arise because biological reality is messy and the underlying processes complex. Mathematical models allow us to examine the consequences of a set of clearly defined assumptions and to assess their relative importance in governing a biological pattern of interest (Servedio et al. 2014). Converting verbal models to mathematical ones means that any unstated (or unrecognized) assumptions come to the forefront, which can highlight contradictions or unexpected interactions.

Too much emphasis on formal models, however, may result in diminishing returns. When Joe Felsenstein reviewed a festschrift put together in honor of the mathematical population geneticist Samuel Karlin, he recounted an anecdote involving Karlin and John Maynard Smith (Felsenstein 1989). The encounter took place at the 1973 International Congress of Genetics, held in Berkeley, California. This was a time when Karlin was dividing his time between Stanford University in the USA and the Weizmann Institute in Israel. During his talk, Maynard Smith presented a solution to an evolutionary problem, a solution that lacked formal rigor and instead relied on approximations. He then paused and said:

…someone like Sam Karlin would never approve of it. However I used to design airplanes for a living, and I can assure Professor Karlin that the very airplanes on which he flies back and forth with such confidence were designed by the very methods he deplores.

—J. Maynard Smith (quoted in Felsenstein 1989)

The methods of Maynard Smith that Karlin deplored were the cruder ones of the original generation of population geneticists—Fisher, Wright, and Haldane—and continued by Maynard Smith. Haldane once said that their mathematical models “may impress zoologists but do not greatly impress mathematicians” (Haldane 1964). Instead, their methods often relied on intuition and approximations. Felsenstein contrasts this original approach with what he calls the Stanford school of thought, represented by researchers like Karlin and Feldman. The Stanford school treated population genetics as a branch of applied mathematics and argued that this provided a more rigorous approach than what they regarded as mere approximations (see also Schwartz 2002).

Karlin and his colleagues raised the bar for what constitutes a model in theoretical population genetics, but to what end? As noted by Felsenstein, and exemplified by our discussion of models of imprinting, an increased focus on rigor is not always the best approach to solve real biological problems. In particular, the evolutionary insights from a model that focuses solely on whether a specific allele will invade or not will be limited if they lose sight of where the selective action is. Identifying that action is exactly what agential thinking is best suited for (Wilson 2005, chapter 4). At their worst, excessively rigorous models result only in what the mathematician Mark Kac called “dehydrated elephants”—impressive achievements of limited relevance (retold in Bari Kolata 1975). Raising similar issues in the discussion about how to model inclusive fitness, Grafen (2009) suggested that it is often useful to abandon the “gold standard” of population genetics in favor of a more practical “plastic standard”: “applied usefulness rather than decoration”.

Others have warned that “fear of the dangers of anthropomorphism has caused ethologists to neglect many interesting phenomena, and it has become apparent that they could afford a little disciplined indulgence” (Hinde 1982, pp. 77–78). The key question is not necessarily if selfish genetic elements are agents, rather than, say, broken cogs in an otherwise well-oiled machine, but whether there is a meaningful way in which they can be described as such (Hammerstein and Hagen 2006; Veit 2021). We consider it clear that they not only can, but that this is a productive way to think about genetic conflicts.

We further believe that the choice need not be between anthropomorphic agential thinking or formal population genetics, but that the two approaches can be combined in a form of licensed anthropomorphism. In his comprehensive book on anthropomorphism in the study of animal behavior, J.S. Kennedy distinguishes between two kinds: genuine and mock anthropomorphizing (Kennedy 1992). The key difference is that whereas the naive ascription of human emotions to non-human entities, what Kennedy calls genuine anthropomorphizing, has little to offer biology, mock anthropomorphism can be very valuable (Kennedy 1992, p. 94; see also Dawkins 1995). Kennedy’s mock anthropomorphism is closely related to Daniel Dennett’s intentional stance:

Here is how it works: first you decide to treat the object whose behavior is to be predicted as a rational agent; then you figure out what beliefs that agent ought to have, given its place in the world and its purpose. Then you figure out what desires it ought to have, on the same considerations, and finally you predict that this rational agent will act to further its goals in the light of its beliefs. A little practical reasoning from the chosen set of beliefs and desires will in most instances yield a decision about what the agent ought to do; that is what you predict the agent will do.

—D. Dennett (1987, p. 17)

In other words: what is a single selfish gene trying to do? We prefer the term “licensed anthropomorphizing” over both mock anthropomorphism and the intentional stance.

The concept of a licensed anthropomorphism, along with the term itself, was first introduced by Alan Grafen at a conference organized by the Royal Statistical Society in conjunction with the unveiling of a plaque to commemorate R.A. Fisher at Inverforth House, Hampstead, London in 2002 (later published as Grafen 2003). Grafen had been invited to talk about Fisher’s contribution to evolutionary theory, a task he took up with Gusto (describing himself as “a Fisher fanatic [who believes] him to have been right about nearly everything in statistics, genetics, and evolutionary biology”). In particular, Grafen argues that Fisher’s fundamental theorem of natural selection (the much-misunderstood cornerstone of Fisher’s work on evolutionary biology that suggests that the rate of increase in population mean fitness that is due to selection causing changes in allele frequencies will be equal to the genic variance in fitness) makes it defensible to talk about organisms as trying to maximize their fitness. This is the premise of his Formal Darwinism Project (Grafen 2014a, b; Okasha and Paternotte 2014). We think that this approach can be extended to any biological agent, including genes involved in genetic conflicts.

A licensed anthropomorphizing combines creativity and rigidity. A biologist applying licensed anthropomorphizing will consider the object under study (say, an X chromosome) and ask: if I were a gene on this X chromosome, what would I do to maximize my chances of transmission in this situation? To check the argument, and to expose any weaknesses in its logic, she then develops a mathematical model of her hypothesis. This act of formalization provides the license that makes the anthropomorphizing legitimate. In so doing, it also provides a bridge between what Yanai and Lercher (2019) called “night science”, the creative thought process that occurs after-hours and the more structured and formal “day science” that takes place in the lab and the field. Reciprocally, such agential thinking may also help interpret novel, counterintuitive results, uncovered during the formal modeling of a problem.

Without using the term itself, Maynard Smith summarized the benefits of a licensed anthropomorphism well:

I am prepared to think as loosely as necessary to give me an idea when I’m confronted with a new biological problem. If it helps me think to say, ‘If I was a gene, I would do so-and-so’, then I think that is OK. But when I’ve got an idea, I want to be able to write down the equations and show that the idea works. (...) I’m all for loose thinking. We all need ideas.

—J. Maynard Smith (1998)

Not all problems, however, can be solved with a licensed anthropomorphism. Anthropomorphizing genes may also distract from alternative explanations. Focusing on the selfishness of individual genes may lead to an over reliance on conflict explanations (Wade and Drown 2016). For example, the licensed anthropomorphism of a gene’s-eye view combined with theoretical population genetics offers an intuitive and well-supported explanation for how and why uniparentally inherited mitochondrial genes and biparentally inherited nuclear genes come into conflict (Hurst and Hamilton 1992). The same transmission argument, however, ought to apply to the uniparentally inherited chloroplast genome. Yet such conflicts seem to be vanishingly rare (though see Sobanski et al. 2019 for an example).

Evolutionary biology as a scientific discipline is characterized by both uniformitarianism and exceptionalism. On the one hand, the principles of evolution by natural selection apply equally in all organisms—what is true for *E. coli* is true for the elephant, as Monod put it. On the other, evolutionary biology is a messy historical science and the properties of living systems can rarely be derived from first principles, even in the simplest of cases.

## Conclusion

Recent years have demonstrated the power of many tools first developed in behavioral ecology, as the study of genetic conflict so clearly illustrates. That is not to say that the tools are without critics, a fact also clearly illustrated by the study of genetic conflicts, which has seen disparate research traditions in evolutionary biology coalesce around the same phenomena. The behavioral ecology tradition has been comfortable with anthropomorphic agential thinking and maximization approaches, whereas formal population genetics, especially in the tradition of Karlin and Feldman, have eschewed and dismissed such thinking. Here, we have demonstrated that the strengths of the warring strategies can be combined in a licensed anthropomorphism that allows the benefit of both to be maximized.
